# Hand, foot, and mouth disease reinfection in Quzhou, China: A 16-year retrospective cohort study of epidemiology and risk factors

**DOI:** 10.1097/MD.0000000000045310

**Published:** 2025-10-17

**Authors:** Quanjun Fang, Jilai Mao, Shuangqing Wang, Xiaoying Gong, Canjie Zheng, Zhiying Yin

**Affiliations:** a Department of Immunity, Quzhou Center for Disease Control and Prevention, Quzhou, Zhejiang, China; b Department of Immunity, Jiangshan Center for Disease Control and Prevention, Jiangshan, Zhejiang, China.

**Keywords:** cox proportional hazards model, epidemiological characteristic, hand, foot, and mouth disease, reinfection, risk factors

## Abstract

Previous studies on hand, foot, and mouth disease (HFMD) have primarily addressed initial infections, with limited focus on reinfection trends and multifactorial risk interactions. Conventional methods like logistic regression often fail to capture temporal risk dynamics. This study applied a Cox proportional hazards model to analyze epidemiological traits and risk factors for HFMD reinfection in Quzhou from 2008 to 2024, to guide prevention strategies. Among 79,841 reported cases, 3945 reinfections were identified, yielding a reinfection rate of 4.94%. Most cases experienced 2 episodes (96.53%), with fewer having 3 (3.40%) or 4 (0.08%). Infections showed bimodal seasonal peaks in April–June and November–December. Median intervals between successive infections decreased from 1.38 years (1st to 2nd) to 0.82 years (3rd to 4th). Cox proportional hazards regression modeling identified age under 3 years, scattered children, urban residence, initial infection with coxsackievirus A16, and coxsackievirus A16 being the predominant circulating strain in the year of initial infection as significant risk factors for HFMD reinfection. our study identifies a significant HFMD reinfection burden (4.94%) in Quzhou. These findings highlight the need for targeted health education and sustained vigilance, especially for high-risk groups, to mitigate reinfection risks. However, only 4.16% of reinfection cases in this study were laboratory-confirmed, and the diagnostic reagents did not permit differentiation across all serotypes. Variation in detection rates among reinfection cases may have introduced bias, and the related findings should therefore be interpreted with caution.

## 1. Introduction

Hand, foot, and mouth disease (HFMD) is a category C notifiable infectious disease caused by infection with multiple enteroviruses. It requires routine monitoring and reporting and is generally regarded as having a lower public health impact than category A and B diseases. The condition predominantly affects children under 5 years of age,^[1]^ with enterovirus 71 (EV-A71) and coxsackievirus A16 (CV-A16) being the most common causative pathogens. The majority of patients with HFMD exhibit a mild, self-limiting illness, typically characterized by a vesicular rash on the hands, feet or buttocks, accompanied by oral ulcers or blisters, with or without fever.^[2]^ This classic clinical presentation, along with its atypical variants, has been consistently observed in recent studies.^[3]^ However, a subset of patients may progress to severe disease, and in extreme cases, the condition can be fatal.^[4]^

Since the 1990s, numerous outbreaks of HFMD have been reported across countries in the Western Pacific region,^[5,6]^ contributing to a substantial disease burden.^[7]^ HFMD has therefore become an increasingly pressing public health concern. In China, HFMD has occurred annually across much of the country since it was 1st identified in Shanghai in 1981.^[8]^ In 2008, a major outbreak in Fuyang City, Anhui Province, resulted in 22 fatalities.^[9]^ That same year, the Chinese Ministry of Health classified HFMD as a category C notifiable infectious disease. In recent years, the average annual number of reported HFMD cases in mainland China has exceeded 1 million,^[10]^ ranking 1st among all notifiable infectious diseases.^[11]^ As such, HFMD has emerged as a major public health concern.

Vaccination remains the most cost-effective strategy for the prevention of infectious diseases. Since its introduction to the mainland Chinese market in 2016, the EV-A71 vaccine has been widely adopted. Evidence indicates^[12]^ that a 2-dose schedule confers substantial effectiveness in real-world settings, reducing both the incidence of EV-A71-associated HFMD and the occurrence of severe cases. However, the vaccine offers protection solely against EV-A71 and does not confer cross-protection against other enteroviruses. Moreover, the antibody response elicited by the EV-A71 vaccine is not lifelong, and reinfection remains possible.^[13]^ As a result, HFMD reinfection is not rare. Reinfection can lead to more complex symptoms, including atypical rashes and bacterial coinfections, and may heighten the disease burden because the immune response to different serotypes varies.^[14]^ Epidemiological data indicate a rising trend in HFMD reinfection rates, potentially attributable to pathogen diversity and the transient nature of host immune memory.^[15,16]^ For instance, reinfection cases reported in Saudi Arabia suggest that insufficient cross-immunoprotection against different viral serotypes, or antigenic drift, may be key factors driving reinfection.^[17]^ Additionally, host genetic factors and immunosuppressive conditions may affect the risk of reinfection by modulating the efficiency of viral receptor binding or the antibody response.^[18,19]^

Previous studies have predominantly focused on primary infections, with limited systematic analyses of the temporal patterns of reinfection and the interplay of multidimensional risk factors. Conventional analytical approaches, such as logistic regression, struggle to effectively capture risks associated with temporal dynamics. This gap necessitates more robust statistical techniques. In particular, survival analysis models that account for time-dependent variables have been seldom used in studying HFMD reinfection. In this study, a Cox proportional hazards regression model was employed, with reinfection as the event of interest and the interval from initial infection to reinfection as the survival time. The effects of multiple factors on survival time were analyzed to elucidate the prevalence characteristics of HFMD and identify risk factors for reinfection in Quzhou City between 2008 and 2024. These findings aim to inform strategies for the prevention and control of HFMD.

## 2. Materials and methods

### 2.1. Study design

This study employed a retrospective cohort design to analyze the epidemiological characteristics and risk factors associated with HFMD reinfection in Quzhou City from 2008 to 2024.

### 2.2. Data collection

HFMD cases were obtained from the Chinese Information System for Disease Control and Prevention, with data extracted based on current address and date of onset, and only cases with verified information were included. Our study data encompassed all laboratory-confirmed and clinically diagnosed HFMD cases reported between 2008 and 2024, with ages ranging from 0 to 70.05 years. In this research, HFMD predominantly affected children under 5 years of age (90.11%). Including cases across all age groups ensured data completeness and avoided selection bias. Population classification was based on the child’s care setting. Scattered children: Not enrolled in any educational institution and typically cared for at home. Kindergarten children: attending kindergarten. Students: attending primary or secondary school. This classification is consistent with the demographic categories employed in the Chinese Information System for Disease Control and Prevention. Data were collected from all 6 counties (cities/districts) of Quzhou City: Kecheng District, Qujiang District, Jiangshan City, Kaihua County, Longyou County, and Changshan County. The 6 counties (cities and districts) of Quzhou were categorized into urban and rural areas based on geographic distribution, with Kecheng District and Qujiang District classified as urban, and the remaining areas as rural. Disease severity was classified according to the Clinical Guidelines for Hand, Foot and Mouth Disease (2018 edition)^[20]^ issued by the Chinese National Health Commission – mild cases: presented with rash, vesicles, or oral ulcers, with or without fever. Severe cases: presented with neurological (e.g., meningitis, encephalitis) or cardiopulmonary complications (e.g., pulmonary edema, heart failure). Additionally, pathogenetic findings from previous HFMD sentinel surveillance in Quzhou City were incorporated.

### 2.3. Case definitions

The diagnostic criteria and severity classification for HFMD were based on the Clinical Diagnosis and Treatment Guidelines for Hand, Foot and Mouth Disease (2018 Edition) issued by the Chinese Ministry of Health.^[20]^ A clinically diagnosed case was defined by the presence of a vesicular rash on the hands, feet, mouth or buttocks, with or without fever. A laboratory-confirmed case was defined as a clinically diagnosed case accompanied by laboratory evidence of enterovirus infection (EV-A71, CV-A16, or other enteroviruses), confirmed through reverse transcription-polymerase chain reaction (RT-PCR) or viral isolation. According to these guidelines, cases were classified as severe only if clinical or laboratory diagnosis revealed the development of neurological complications (e.g., aseptic meningitis, encephalitis, acute flaccid paralysis), cardiopulmonary complications (e.g., pulmonary edema, pulmonary hemorrhage, cardiogenic shock), or both. All cases that did not meet these criteria for severe disease were uniformly classified as mild. This dichotomous classification is the standard and mandated approach for HFMD surveillance in China, and no moderate category is defined in the national guidelines. HFMD reinfection cases were defined as individuals infected with HFMD 2 or more times during the period from 2008 to 2024. If the patient’s initial HFMD episode was classified as mild, any subsequent episode occurring within 14 days of the 1st was considered a duplicate report. A recurrence with an interval of 14 days or more was defined as a reinfection. If the patient’s initial HFMD episode was classified as severe, any subsequent episode occurring within 23 days of symptom onset was considered a duplicate report. An interval of 23 days or more was defined as reinfection. These interval thresholds were adopted from the methodology established by Huang et al, which were derived by adding the longest reported duration of HFMD symptoms (7 days for mild cases and 16 days for severe cases) to the longest reported incubation period (7 days) to conservatively differentiate between prolonged viral shedding and a true reinfection event.^[21]^

### 2.4. Case classification

Cases in this study were categorized as either clinically diagnosed or laboratory-confirmed. Laboratory-confirmed cases were defined as those in which anal swab or fecal specimens tested positive by colloidal gold immunochromatography or RT-PCR. Confirmed cases were classified by viral serotype as EV-A71, CV-A16, or other enteroviruses. Colloidal gold testing was performed by laboratories across all levels of medical institutions in Quzhou City. RT-PCR testing was performed by the laboratory of the Quzhou Centre for Disease Control and Prevention. All reagents used were commercially available and fully certified.

### 2.5. Data cleaning

The system exported 80,050 HFMD case records, of which 79,841 remained after exclusion of suspected cases and duplicate reports. To ensure accurate identification of reinfection cases, we defined the following inclusion and exclusion criteria. Construction of the preliminary screening pool. Case records meeting any of the following criteria were included in the preliminary pool: matching identification number; or matching parental names and patient date of birth; or matching telephone number and patient date of birth. Application of these criteria yielded an initial pool of 8364 case records, corresponding to 4088 unique individuals. The initial pool of 4088 unique individuals was then subjected to uniform exclusion criteria: names not sufficiently similar; failure to meet the definition of a reinfection interval (<14 days if the initial illness was mild, <23 days if severe); and residential addresses located in different counties or districts. After applying these criteria, 8030 infection records remained, corresponding to 3945 distinct individuals.

### 2.6. Ethical approval

This study was approved by the Research Ethics Committee of the Centre for Disease Control and Prevention of Quzhou City (approval number: 2025-006-02). The committee waived the requirement for individual informed consent for the following reasons: the study utilized anonymized surveillance data collected under the Law of the People’s Republic of China on Prevention and Control of Infectious Diseases; all personally identifiable information was removed prior to analysis; and secondary analysis of such anonymized public health data is exempt from ethical review according to the National Health Commission of China.

### 2.7. Methods

Descriptive epidemiological methods were employed to analyze the timing of reinfection onset, population distribution, regional distribution, and etiological characteristics. Cox proportional hazards regression, a widely used method in medical survival analysis, was employed in this study to investigate the risk factors associated with HFMD reinfection. Cumulative risk probabilities were estimated and plotted, and subgroup differences in survival curves were assessed using the log-rank test. In this study, survival time was defined as the interval between the 1st infection and subsequent reinfection; for individuals with 3 or 4 episodes, only the interval between the 1st and 2nd infections was considered. To assess the impact of the predominant strain during the year of initial HFMD infection on the risk of reinfection, data from previous pathogen surveillance in Quzhou City were utilized. EV-A71 was identified as the predominant strain in 2009, 2019, 2022, and 2024; CV-A16 in 2010, 2011, and 2018; while other enterovirus types predominated in the remaining years. The dominant strain in the year of 1st infection was incorporated into the model analysis as the epidemic strain variable. Cox proportional hazards regression was used to calculate hazard ratios (HR), where an HR > 1 indicates that the exposure is a risk factor for the event of interest, while an HR < 1 suggests a protective effect.

### 2.8. Statistical analysis

Data were compiled using Microsoft Excel 2010, and statistical analyses were performed using SPSS version 27.0 (IBM Corp., Armonk). The HFMD reinfection rate was calculated as follows: reinfection rate = (number of HFMD reinfection cases/total number of HFMD cases) × 100%. Continuous variables were described using medians and interquartile ranges (IQR), while categorical variables were summarized using proportions and rates. The Kaplan–Meier method was applied to stratify and estimate the cumulative risk probability of 1st HFMD reinfection by sex, age, population type, region, symptom severity, and the predominant strain in the year of initial infection. Differences were assessed using the log-rank test. Cox proportional hazards regression models were utilized for univariate and multivariate analyses of potential risk factors influencing reinfection. A significance level of α = 0.05 was adopted. The Cox proportional hazards model was employed to evaluate individual-level risk factors for reinfection. Population-level and time-varying factors, such as season, month, or meteorological conditions, which affect all subjects uniformly within a given time period, were not included as covariates in the model. The primary aim was to identify host-specific risks rather than to model the external environmental drivers of HFMD epidemics, which are already well-established.^[22]^

## 3. Results

### 3.1. Epidemiological characteristics of reinfection cases

Between January 2008 and December 2024, a total of 3945 cases of HFMD reinfection were reported in Quzhou, representing a reinfection rate of 4.94% (3945/79,841). Among them, 2468 were male, with a reinfection rate of 5.30% (2468/46,552), and 1477 were female, with a reinfection rate of 4.44% (1477/33,289). The difference in reinfection rates between sexes was statistically significant (χ^2^ = 31.686, *P* < .001). Most individuals (3808 cases; 96.53%) experienced 2 infections, while 134 cases (3.40%) experienced 3 infections and 3 cases (0.08%) experienced 4 infections. By region, the number of reinfection cases was highest in Kecheng District (1378; 34.93%), followed by Qujiang District (750; 19.01%), Kaihua County (624; 15.82%), Longyou County (476; 12.07%), Changshan County (419; 10.62%), and Jiangshan City (298; 7.55%). The corresponding reinfection rates were 5.80% (750/13,120) in Qujiang District, 5.64% (1378/24,451) in Kecheng District, 5.20% (624/11,996) in Kaihua County, 4.60% (419/9113) in Changshan County, 3.97% (476/11,993) in Longyou County, and 3.25% (298/9168) in Jiangshan City. Severe cases accounted for 0.10% (4/3945) during the 1st infection and 0.08% (3/3945) during the 2nd, with no severe cases reported in the 3rd or 4th infections. All 7 severe cases occurred in children.

**Figure 1.**
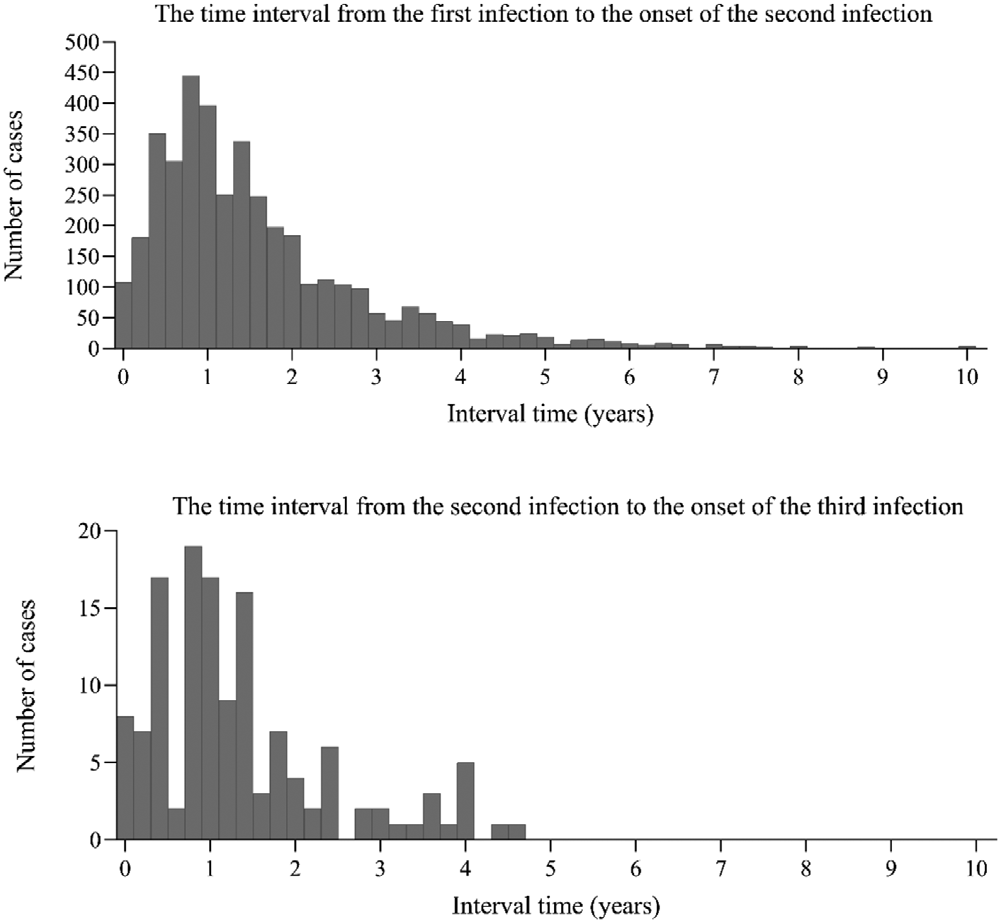
Distribution of time intervals between onset of hand, foot, and mouth disease reinfection episodes in Quzhou, 2008 to 2024.

Comparison of the characteristics of cases with 1st, 2nd, and 3rd to 4th HFMD infections revealed that the median age at infection was 1.69 years (IQR: 1.20–2.66), 3.38 years (IQR: 2.55–4.56), and 3.68 years (IQR: 3.08–5.56), respectively. The intervals between successive infections exhibited a skewed distribution, with median values of 1.38 years (IQR: 0.83–2.14), 1.08 years (IQR: 0.71–1.76), and 0.82 years, respectively. The population classifications were predominantly scattered children (78.15%) for 1st infections, and kindergarten children for both 2nd (53.51%) and 3rd to 4th infections (65.00%). The number of severe cases reported was 4, 3, and 0 for 1st, 2nd, and subsequent infections, respectively. Etiological test results were dominated by EV-A71 across all episodes, accounting for 65.85%, 49.04%, and 84.62%, respectively (Table 1, Fig. 1).

**Table 1:** Epidemiological characteristics of hand, foot, and mouth disease reinfection cases in Quzhou, 2008 to 2024.

Characteristic	1st infection (n = 4026)		2nd infection (n = 4026)		The 3rd to 4th infection (n = 146)	
	n	%	n	%	n	%
Age (yr)						
<0.5	37	0.94	0	0.00	0	0.00
0.5-	547	13.87	20	0.51	0	0.00
1-	1728	43.80	467	11.84	4	2.86
2-	1573	39.87	2783	70.54	95	67.86
5-	59	1.50	656	16.63	41	29.29
≥10	1	0.03	19	0.48	0	0.00
Population classification						
Scattered children	3083	78.15	1653	41.90	39	27.86
Kindergarten children	854	21.65	2111	53.51	91	65.00
Student	8	0.20	181	4.59	10	7.14
Clinical type						
Severe	4	0.10	3	0.08	0	0.00
Mild	3941	99.90	3942	99.92	140	100.00
Laboratory results*						
EV-A71	108	65.85	77	49.04	11	84.62
CV- A16	23	14.02	32	20.38	0	0.00
Other EV	33	20.12	48	30.57	2	15.38

EV-A71 = enterovirus A71, CV- A16 = coxsackievirus A 16.

* The number of cases with pathogen detection during the 1st, 2nd, and 3rd to 6th infections was 164, 157, and 13, respectively. Scattered children are preschool children not attending any childcare facility; kindergarten children are those enrolled in a kindergarten; students are children enrolled in primary school.

### 3.2. Temporal distribution of reinfection cases

**Figure 2.**
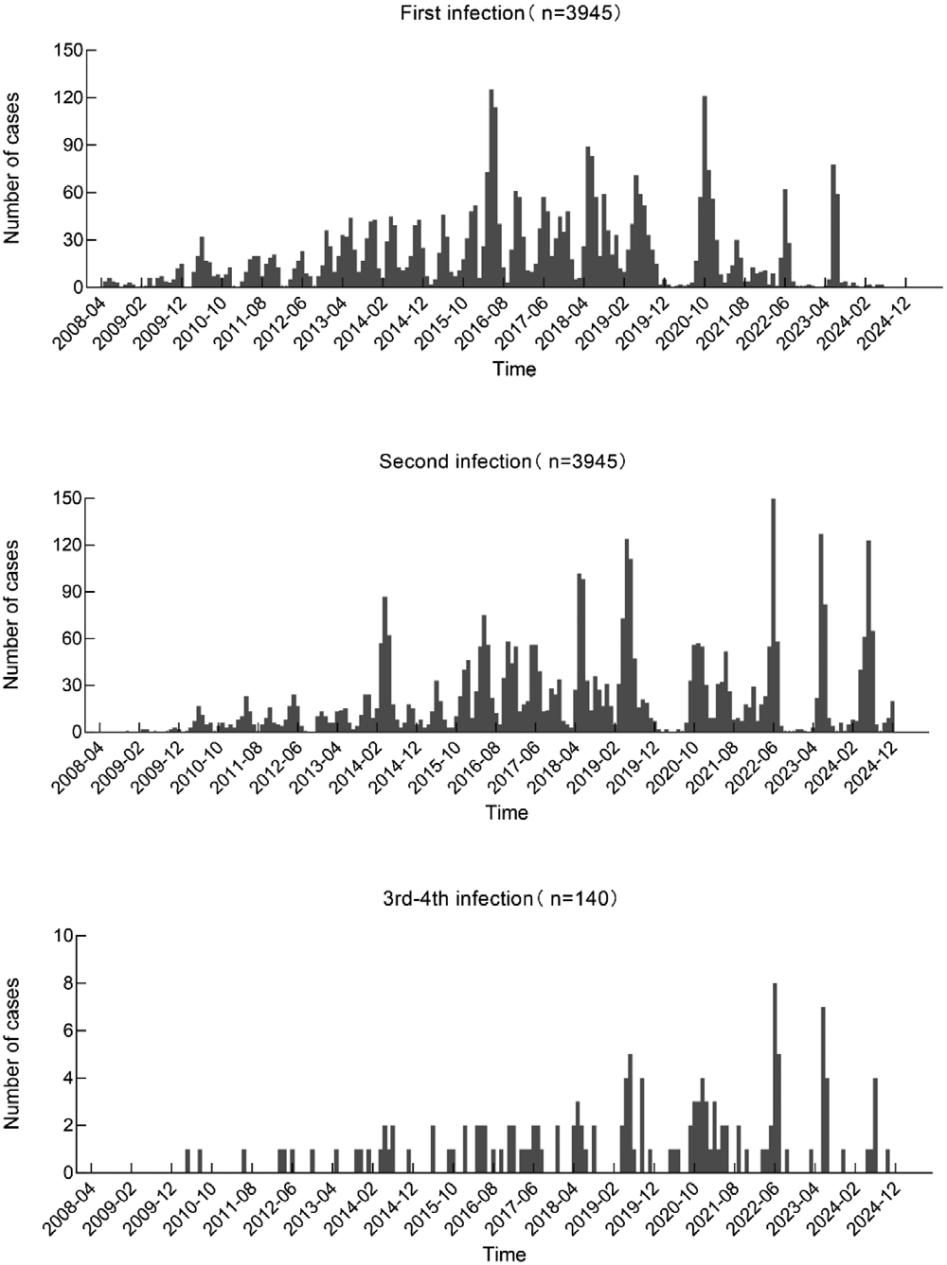
Prevalence curve of reinfection cases of hand, foot, and mouth disease in Quzhou, 2008 to 2024.

There was a clear seasonal pattern in the onset of all 1st to 3rd infections, displaying a bimodal distribution. The 1st peak of incidence occurred between April and June, accounting for 38.40%, 50.36%, and 49.44% of cases, respectively; the 2nd peak occurred between November and December, comprising 19.95%, 14.75%, and 12.23% of cases, respectively (Fig. 2). The 4th infection was reported in 3 cases, with onset occurring in April, June, and July, respectively.

### 3.3. Pathogenetic features of reinfection cases

Among the reinfection cases, 52 had laboratory confirmation for both the 1st and 2nd infections. EV-A71, CV-A16, and other enterovirus types were all capable of causing recurrent infections. The highest proportion of cases involved EV-A71 on both occasions (65.38%), followed by reinfection with CV-A16 after an initial EV-A71 infection (13.46%) and other enterovirus types following EV-A71 infection (11.54%; Table 2). There were 6 confirmed cases of 3rd infection, among which 5 were caused by EV-A71 (with both 1st and 2nd infections confirmed, comprising 2 cases of EV-A71, 2 cases of CV-A16, and 1 case of other enterovirus types), and 1 case was caused by other enterovirus types (with both 1st and 2nd infections clinically diagnosed). All 4th infections were clinically diagnosed cases.

**Table 2:** Aetiology of 1st and 2nd infections in reinfected hand, foot, and mouth disease cases in Quzhou, 2008 to 2024.

Virus of 1st infection	Virus of 2nd infection	n	%
EV-A71	EV-A71	34	65.38
EV-A71	CV-A16	7	13.46
EV-A71	Other EV	6	11.54
CV-A16	CV-A16	2	3.85
CV-A16	Other EV	1	1.92
Other EV	EV-A71	1	1.92
Other EV	CV-A16	1	1.92

Among the reinfection cases, 52 had laboratory confirmation for both the 1st and 2nd infections.

EV-A71 = enterovirus A71, CV- A16 = coxsackievirus A 16.

### 3.4. The hazard of HFMD reinfection

**Figure 3.**
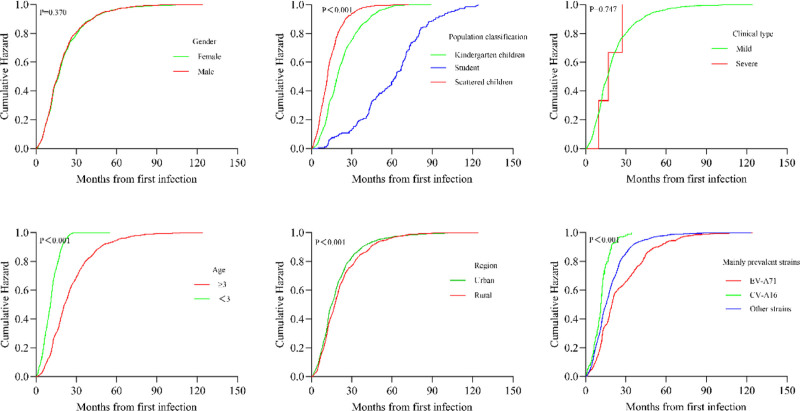
Kaplan–Meier curve depicting the cumulative hazard of hand, foot, and mouth disease reinfection.

The risk of reinfection was significantly elevated within 20 months following the initial HFMD infection, with 25.27% (997/3945) of cases reinfected within 10 months and 62.38% (2461/3945) reinfected within 20 months. The cumulative hazard probability curves for HFMD reinfection, stratified by 6 factors – sex, population category, symptom severity, age group, place of residence, and the predominant strain in the year of 1st infection – indicated a higher risk of reinfection among children under 3 years of age, scattered children, those living in urban areas, and individuals whose 1st infection occurred during a year when CV-A16 was the predominant circulating strain (Fig. 3).

Cox proportional hazards regression analyses, adjusted for relevant covariates, indicated that age group, population classification, region of residence, and the predominant enterovirus strain during the year of primary infection were all significantly associated with the risk of HFMD reinfection, even after accounting for potential confounding variables. Notably, children aged ≥3 years exhibited a substantially lower risk of reinfection compared with those aged <3 years (HR = 0.335, 95% CI: 0.258–0.344). Compared with scattered children, those in kindergarten care and school students had a significantly lower risk of reinfection (HR = 0.722, 95% CI: 0.656–0.823; and HR = 0.122, 95% CI: 0.095–0.145, respectively; Table 3).

**Table 3:** Cox proportional hazards regression analysis of factors influencing hand, foot, and mouth disease reinfection in Quzhou, 2008 to 2024.

Characteristic		Single-variable analysis			Multivariable analysis		
		HR	95% CI	*P*	HR	95% CI	*P*
Gender	Female						
	Male	1.030	0.966–1.099	.368	1.044	0.925–1.122	.519
Age(Years)	<3						
	≥3	0.241	0.223–0.260	<.001	0.335	0.258–0.344	<.001
Population classification	Scattered children						
	Kindergarten children	0.481	0.450–0.515	<.001	0.722	0.656–0.823	<.001
	Student	0.082	0.068–0.100	<.001	0.122	0.095–0.145	<.001
Region	Rural						
	Urban	1.140	1.070–1.213	<.001	1.109	1.042–1.185	<.001
Clinical type	Severe						
	Mild	1.175	0.378–3.652	.781	1.203	0.256–3.349	.658
Prevalent strain in year of 1st infection	EV-A71						
	CV-A16	3.144	2.651–3.621	<.001	2.365	2.015–2.811	<.001
	Other EV	1.556	1.451–1.864	<.001	1.323	1.254–1.527	<.001
Laboratory results*	EV-A71						
	CV-A16	2.407	1.253–5.815	.015			
	Other EV	0.389	0.292–1.265	.315			

Scattered children are preschool children not attending any childcare facility; kindergarten children are those enrolled in a kindergarten; students are children enrolled in primary school.

EV-A71 = enterovirus A71, CV-A16 = coxsackievirus A 16, HR = hazard ratios.

* Among the 3945 reinfection cases, only 164 were confirmed by laboratory testing. Consequently, the “pathogen” variable contains numerous missing values and was excluded from the multivariate analysis.

## 4. Discussion

HFMD is a common infectious illness in children, responsible for numerous clinical cases annually and posing a risk of mortality in severe instances.^[23]^ Despite growing parental awareness and prevention efforts, reinfection remains a significant threat to children’s health. The HFMD reinfection rate in Quzhou City was 4.94%, exceeding the 3.07% reported in Guangzhou from 2012 to 2017^[24]^ and the national average of 3.25% from 2008 to 2015,^[21]^ remaining below the 5.48% observed in Jiulongpo District, Chongqing from 2009 to 2023.^[25]^ Discrepancies in reinfection rates across studies likely arise from differences in study periods, reinfection definitions, and regional socio-demographic factors like economic development and population density. These findings also indicate that the reinfection rate of HFMD in Quzhou remains high, emphasizing the urgent need for further research into the risk of reinfection.

The HFMD reinfection rate varied across counties, cities and districts within Quzhou City, ranging from 3.25% to 5.80%. This suggests potential spatial heterogeneity in reinfection rates, the underlying causes of which require further investigation. HFMD reinfections predominantly involved 2 episodes, with cases of 3 or more infections being less common, consistent with previous studies.^[21]^ Multiple HFMD infections predominantly occurred in children aged under 4 years, with median ages of 3.38 and 3.68 years for the 2nd and 3rd infections, respectively. Correspondingly, the population categories for the 1st to 3rd infections were primarily composed of children living in scattered settings for the initial infection, followed by those in early childhood care for the 2nd and 3rd infections. A marked seasonality was evident in the onset of HFMD reinfection cases, with the seasonal patterns of the 1st, 2nd and 3rd infections being largely consistent. The primary peak in incidence occurred between April and June, consistent with the findings of Tian et al^[26]^ However, this study also identified a secondary peak during November–December. Xu et al^[27]^ reported that the incidence of HFMD was positively associated with ambient temperature, with the strongest correlation observed within the 25.0 to 27.5°C range. From the perspective of the temporal interval between reinfections, the median duration between successive HFMD episodes exceeded 1 year, consistent with findings from Fujian Province.^[28]^ This underscores the importance of disseminating relevant health education to parents and emphasizing the need for vigilance in preventing reinfection within 2 years of the initial episode.

HFMD reinfection can be attributed to a diverse range of enteroviruses. Reinfection with EV-A71, CV-A16, or other enterovirus types may occur following an initial EV-A71 infection, and in some instances, homologous reinfection has been observed. The underlying reasons may be attributed, firstly, to the failure of some patients to develop effective humoral immunity following HFMD virus infection^[29]^; and secondly, to the limited or absent cross-immunity between different enterovirus types.^[30]^ However, the high proportion of recurrent EV-A71 infections following initial EV-A71 infection may be attributable to the fact that EV-A71 testing is primarily conducted in Quzhou City, and therefore, the results may not be generalizable. Furthermore, the incidence of reinfection was higher when the initial infection was caused by CV-A16 or when CV-A16 was the predominant strain in the year of 1st infection. This finding aligns with studies conducted nationwide,^[21]^ as well as in Anhui Province,^[31]^ and may be related to differences in the shedding rates of various subtypes. Fu and coworkers^[32]^ examined the epidemiological trends and age-cohort effects of HFMD in Quzhou City. Their findings show that, since 2018, CV-A16 and CV-A6 have gradually emerged as the predominant strains, indicating an increasing risk of HFMD reinfection in the region.

A review indicated that EV-A71 exhibits slower viral shedding and requires a longer period for complete clearance by the host compared with CV-A16. This prolonged clearance may contribute to the development of more durable immunity against EV-A71 and consequently a reduced risk of reinfection.^[33]^ In this study, it was observed that while reinfection could occur following a severe initial infection, subsequent episodes did not progress to severe disease. Conversely, cases where the 1st infection was mild could still result in reinfection with progression to severe disease. The severity of reinfection may depend on the viral subtype involved, a hypothesis that requires validation in future studies. Both univariate and multivariate analyses indicated that the risk of hand, HFMD reinfection was significantly associated with demographic factors, including age, area of residence and population classification. Notably, being under 3 years of age, residing in urban settings, and belonging to the Scattered children emerged as independent risk factors for reinfection. In a systematic study, Luo et al^[34]^ reported that neonatal antibody levels against EV-A71 and CV-A16 declined to their lowest point by 1 year of age, followed by a gradual increase, peaking at approximately 4 years of age. Zhang et al^[35]^ observed that organismal immunity to enteroviruses in children increases progressively with age. Consequently, reinfection with HFMD is more likely to be observed in children under 3 years of age. This study identified a relatively higher risk of reinfection among children residing in urban areas compared to their rural counterparts. This finding contrasts with the commonly held assumption that superior urban sanitation correlates with a reduced risk of reinfection. It may be attributable to enhanced access to and utilization of healthcare services among urban populations, greater parental awareness regarding the importance of seeking medical care, and consequently, a higher likelihood of initial diagnosis and subsequent re-diagnosis of HFMD in urban children.^[36]^ Moreover, urban areas are characterized by high population density and greater levels of transience, both of which contribute to increased interpersonal proximity and thereby elevate the risk of viral exposure.^[37,38]^ Diaspora children exhibit a higher risk of reinfection compared to those in childcare or other population groups, which may be attributable not only to lower immunization coverage but also to comparatively poorer hygiene practices within this demographic.

Since the nationwide rollout of the EV-A71 inactivated vaccine in June 2016, the serotype-specific incidence of EV-A71-associated HFMD has declined substantially, markedly reducing the burden of severe illness and mortality.^[39]^ However, as the vaccine does not provide cross-protection against CV-A16 or other enteroviruses,^[40]^ the overall incidence of HFMD has remained relatively stable, while the relative proportion of non-EV-A71 serotypes has increased. Therefore, while sustaining efforts to promote EV-A71 vaccination to reduce severe outcomes, public health authorities and clinicians should also manage parental expectations by clearly communicating that vaccinated children may nonetheless acquire HFMD caused by other enteroviruses. We observed that when CV-A16 predominates in the year of primary infection, the risk of reinfection increases. This finding highlights the epidemiological gap left by the current monovalent EV-A71 vaccine and underscores the urgency of developing multivalent vaccines that include CV-A16 as a minimum component.

## 5. Limitations

Our research comes with certain limitations that warrant consideration. Firstly, the pathogenicity laboratory test results for confirmed cases were obtained from the infectious disease reporting information system, where the detection reagents limited the scope of pathogen identification. The test results were predominantly for EV-A71, with fewer detections of CV-A16, and no further subtyping was performed for other enterovirus strains. This uneven rate of pathogen testing among confirmed cases may have introduced bias into the results. Secondly, we did not rigorously define or follow up on whether initial infections occurred prior to 2008. Consequently, the risk of reinfection may have been underestimated in this study. Thirdly, only a small proportion of reinfection cases (4.16%) were laboratory-confirmed, which may introduce misclassification bias. This represents an inherent limitation of our retrospective study based on the national surveillance system, as virological testing is not performed for all HFMD cases and is dependent on resource availability and clinical indications. Some clinically diagnosed recurrent cases may in fact represent other exanthematous diseases, potentially resulting in an overestimation of true reinfection rates.

## 6. Conclusion

In conclusion, our study identifies a significant HFMD reinfection burden (4.94%) in Quzhou, characterized by a bimodal seasonal pattern and key risk factors including young age (<3 years), urban residence, scattered children status, and CV-A16 infection – particularly when this serotype was predominant. These findings highlight reinfection as a substantive public health concern, urging targeted interventions for high-risk groups and underscoring the need for multivalent vaccines to address the limited coverage of the current EV-A71 immunization. Limitations, such as low laboratory confirmation rates (4.16%) and incomplete serotype coverage in diagnostics, warrant cautious interpretation of these results.

## Acknowledgments

We thank the Quzhou Centre for Disease Control and Prevention, the local community hospitals for their assistance in conducting the testing, and Mao Jilai and Wang Shuangqing for their valuable advice on data analysis.

We confirm that all individuals named in this Acknowledgments section have provided their permission to be listed.

## Author contributions

**Conceptualization:** Jilai Mao, Shuangqing Wang, Zhiying Yin.

**Investigation:** Jilai Mao, Shuangqing Wang, Xiaoying Gong, Canjie Zheng.

**Methodology:** Xiaoying Gong.

**Validation:** Canjie Zheng, Zhiying Yin.

**Funding acquisition:** Zhiying Yin.

**Writing – original draft:** Quanjun Fang.

**Writing – review & editing:** Quanjun Fang.
